# Selection of Functional Quorum Sensing Systems by Lysogenic Bacteriophages in *Pseudomonas aeruginosa*

**DOI:** 10.3389/fmicb.2017.01669

**Published:** 2017-08-31

**Authors:** Miguel A. Saucedo-Mora, Paulina Castañeda-Tamez, Adrián Cazares, Judith Pérez-Velázquez, Burkhard A. Hense, Daniel Cazares, Wendy Figueroa, Marco Carballo, Gabriel Guarneros, Berenice Pérez-Eretza, Nelby Cruz, Yoshito Nishiyama, Toshinari Maeda, Javier A. Belmont-Díaz, Thomas K. Wood, Rodolfo García-Contreras

**Affiliations:** ^1^Department of Microbiology and Parasitology, Faculty of Medicine, National Autonomous University of Mexico Mexico City, Mexico; ^2^Departamento de Genética y Biología Molecular, Centro de Investigación y de Estudios Avanzados del Instituto Politécnico Nacional Mexico City, Mexico; ^3^Institute of Computational Biology, Helmholtz Zentrum München, Deutsches Forschungszentrum für Gesundheit und Umwelt (GmbH) Neuherberg, Germany; ^4^Mathematical Modeling of Biological Systems, Zentrum Mathematik, Technical University of Munich Garching, Germany; ^5^Centro de Ciencias Genomicas, National Autonomous University of Mexico Cuernavaca, Mexico; ^6^Department of Biological Functions Engineering, Kyushu Institute of Technology Kitakyushu, Japan; ^7^Departamento de Bioquímica, Instituto Nacional de Cardiología Mexico City, Mexico; ^8^Department of Biochemistry and Molecular Biology, Pennsylvania State University, University Park PA, United States

**Keywords:** public goods, social cheating, phage therapy, quorum sensing, virulence

## Abstract

Quorum sensing (QS) in *Pseudomonas aeruginosa* coordinates the expression of virulence factors, some of which are used as public goods. Since their production is a cooperative behavior, it is susceptible to social cheating in which non-cooperative QS deficient mutants use the resources without investing in their production. Nevertheless, functional QS systems are abundant; hence, mechanisms regulating the amount of cheating should exist. Evidence that demonstrates a tight relationship between QS and the susceptibility of bacteria against the attack of lytic phages is increasing; nevertheless, the relationship between temperate phages and QS has been much less explored. Therefore, in this work, we studied the effects of having a functional QS system on the susceptibility to temperate bacteriophages and how this affects the bacterial and phage dynamics. We find that both experimentally and using mathematical models, that the lysogenic bacteriophages D3112 and JBD30 select QS-proficient *P. aeruginosa* phenotypes as compared to the QS-deficient mutants during competition experiments with mixed strain populations *in vitro* and *in vivo* in *Galleria mellonella*, in spite of the fact that both phages replicate better in the wild-type background. We show that this phenomenon restricts social cheating, and we propose that temperate phages may constitute an important selective pressure toward the conservation of bacterial QS.

## Introduction

Bacterial quorum sensing (QS) coordinates the expression of cooperative behaviors including the production of costly exoproducts like exoenzymes and siderophores (Popat et al., 2015). This benefits neighbor cells in the population whether they invested in their production or not; hence, these exoproducts are public goods. Given that the production of public goods is costly, individuals that take advantage of them without contributing to their production are social cheaters (Diggle et al., 2007) with the potential to invade the population, causing a tragedy of the commons, which eventually can lead to an extinction of public good producers (Sandoz et al., 2007). However, in nature, QS systems regulating public goods are widespread and conserved among several bacterial species. Therefore, mechanisms that counteract the effects of social cheaters should exist.

One mechanism to reduce cheating is growth of bacteria in environments that promote the physical separation of cooperators and cheaters, decreasing the cheater’s fitness (Hense et al., 2007; Mund et al., 2016). This occurs in highly viscous medium that limits diffusion of public goods (Kummerli et al., 2009). Other known mechanisms that limit cheating are growth under the stress created by compounds such as H_2_O_2_ (García-Contreras et al., 2015) and HCN (Wang et al., 2015) that select the cooperators. Moreover, predation by the protist *Tetrahymena pyriformis* strongly selects the QS wild-type phenotype of *Pseudomonas aeruginosa*, likely due to the impact of QS on aggregation and biofilm formation (Friman et al., 2013).

A common source of stress for bacteria is the challenge of bacteriophages, which are by far the most abundant biological entities, surpassing bacterial populations by a factor of 10 (Chibani-Chennoufi et al., 2004). Evidence that QS signaling may be involved in regulating the response to phages is increasing. For example, *Escherichia coli* is able to sense QS signals produced by other bacterial species (Michael et al., 2001). In the presence of QS signals, *N*-acyl-L-homoserine lactones, the bacterium significantly reduces the number of phage receptor LamB (Hoyland-Kroghsbo et al., 2013), which protects it against the attack of the l phage. A similar phenomenon occurs in *Vibrio anguillarum*, since mutants that are permanently locked in a high-cell density state are almost completely immune to the phage KVP40, due the QS-mediated downregulation of the OmpK receptor used by the phage (Tan et al., 2015). This phenomenon contributes to a higher phage attachment and greater killing of the QS mutant locked in a low-cell density state (Tan et al., 2015). Similarly, in *Vibrio cholerae*, it was recently shown that QS protects it against the attack of lytic bacteriophages like JSF35 also by downregulating the phage receptor (LPS O-antigen) and by upregulating the expression of the hemagglutinin protease HAP (Hoque et al., 2016).

For *P. aeruginosa*, an important Gram-negative opportunistic pathogen, it was recently shown that QS decreases infection by the lytic phages K5 and C11 (Qin et al., 2016). However, the lytic phage PT7 decreases the *P. aeruginosa* wild-type population density more than that of an isogenic *lasR* mutant (Mumford and Friman, 2017) yet, the presence of an active *Pseudomonas* quinolone signal (PQS) QS system allows *P. aeruginosa* to grow better in the presence the lytic phages PT7 and 14/1 (Moreau et al., 2017).

Since all the previously mentioned studies were done with lytic phages, we studied here for the first time the differences in the susceptibility of a *P. aeruginosa* wild-type and a QS mutant to the infection of two temperate phages D3112 and JBD30 as well as the ability of those phages to interfere with the sociomicrobiology of *P. aeruginosa* by selecting strains with active QS systems. We demonstrate that despite the fact that these bacteriophages replicate more efficiently in QS-proficient strains, the presence of temperate phage leads to the selection of functional QS systems both *in vitro* (counteracting social cheating) and *in vivo* (using *Galleria mellonella*) and that this *in vivo* selection of strains with functional QS systems increases their virulence toward *G. mellonella*. Additionally, our results suggest that QS systems are required for efficient phage production during infection.

## Results

### D3112 and JBD30 Phage Production Is More Efficient on QS Proficient Strains

**FIGURE 1:**
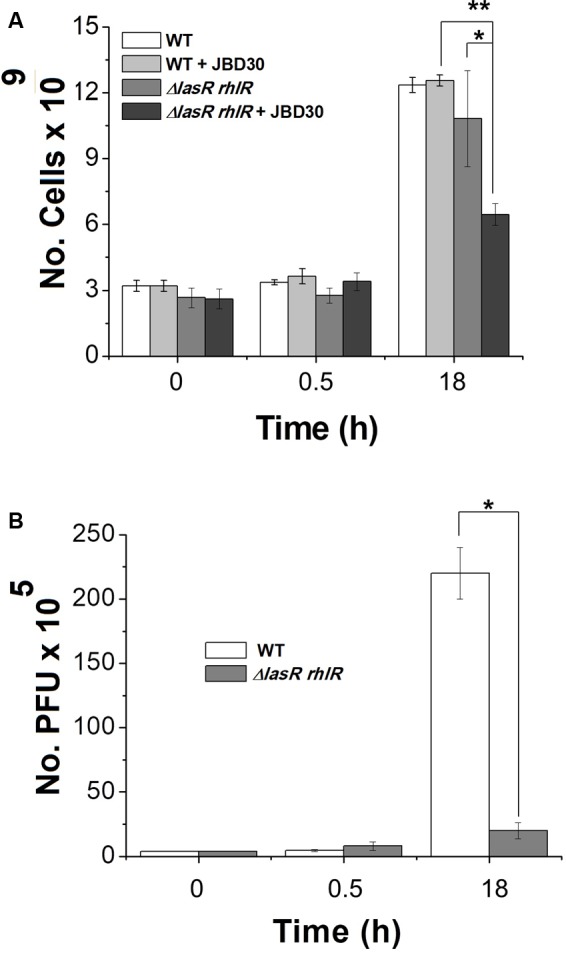
Bacteriophage JBD30 replicates preferentially in the wild-type strain but had a higher killing effect in the QS mutant. Phage JBD30 (2 × 10^6^ pfu) was added to LB cultures of PA14 wild-type and *lasR rhlR* mutant (at OD 600 nm ∼2.2) and the number of cells **(A)** and phage **(B)** were determined at the initial time, and after 0.5 and 18 h after the phage addition. Experiments were done in triplicate and the averages are shown. The cfu of the mutant in the presence of JBD30 at 18 h is significantly lower than the cfu of the mutant without phage (^∗^), as well as the cfu of the mutant in the presence of JBD30 at 18 h, relative to the cfu of the wild-type in the presence of the phage (^∗∗^) *P* < 0.05, in a two-tailed Student’s *t*-test, while the number of phages produced at 18 in the wild-type strain is significantly higher than for the mutant *P* < 0.05, in a two-tailed *t*-test.

D3112 and JBD30 transposable phages were used for this study because they belong to the group D3112 viruses, one of the most ubiquitous groups of *P. aeruginosa* temperate phages (Cazares et al., 2014). We first tested the infection capacity of D3112 and JBD30 on bacterial lawns of the PA14 strain and its QS-defective mutant. In both cases, a greater number of lytic plaques were observed for the PA14 strain as compared to the QS mutant. Phages D3112 and JBD30 produced, in the wild-type strain, about 2.79-fold and 3.28-fold the number of lytic plaques was observed in the mutant, respectively. Accordingly, we observed a tenfold higher phage production of JBD30 on the PA14 strain versus the QS-defective mutant in liquid cultures after 18 h of phage addition, while no change was observed after 30 min (**Figure 1B**). These results show that phage production is more efficient in the wild-type strain and suggest that QS system is required for optimal phage development during infection. Interestingly, JBD30 exerted a significant negative effect on the cell viability of the QS mutant despite that its production of viral particles was poor compared to that observed on the wild-type counterpart after 18 h while no change was observed after 30 min of page addition (**Figure 1A**).

### Selection of Strains with Active QS Systems by Phages D3112 and JBD30 *in Vitro*

Since previous findings relative to the influence of QS in phage infections are based on lytic phage (Tan et al., 2015; Qin et al., 2017), we wanted to test if the presence of the temperate phage could influence competition between the wild-type strain and the QS-deficient mutant. First, the competition experiments were conducted in M9 medium with 1% casamino acids as the carbon source without phage, and it was found that the QS mutant is slightly selected, likely because the QS-deficient mutant does not have the metabolic burden of producing QS products. In contrast, the addition of D3112 or JBD30 bacteriophage selected the wild-type strain after 24 h of co-cultivation (Supplementary Figure S1).

**FIGURE 2:**
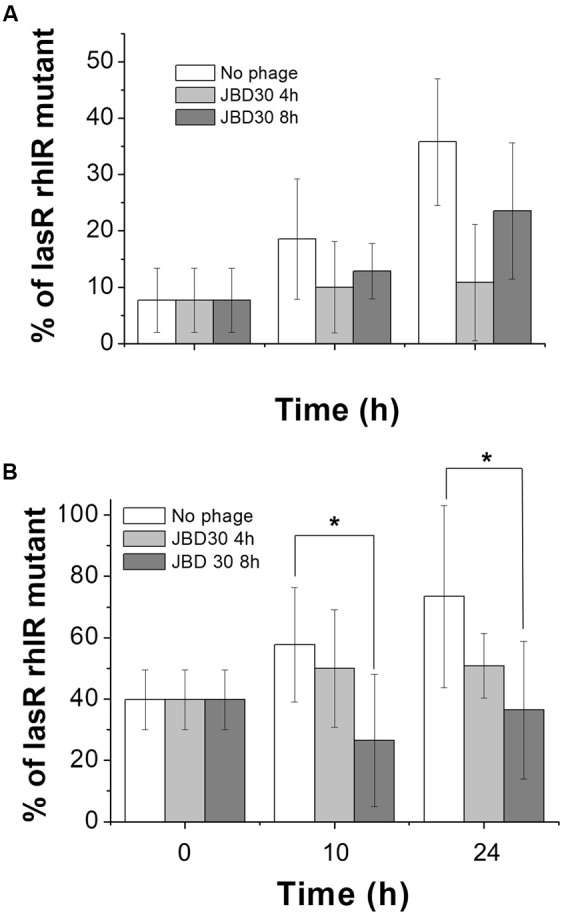
The presence of JBD30 virus decrease social cheating. Competitions were made in M9 caseinate medium. Initial percentages of the *lasR rhlR* mutant were ∼10 **(A)** and ∼50 **(B)**. JBD30 phage (5 × 10^5^ pfu) was added at either 4 h or 8 h after the beginning of the competences. Average of at least three independent cultures is shown. ^∗^Indicate significant differences between experiments without phage and with phage, *P* < 0.01, in a two-tailed Student’s *t*-test.

Additional competition experiments were made in the same medium but supplemented with 0.25% sodium caseinate as the sole carbon source, a condition promoting social cheating. Under this condition, the only way QS-deficient mutants can grow is by using the amino acids and peptides produced by the caseinate hydrolysis mediated by the QS-controlled exoprotease production by the wild-type. Our results confirmed that under such conditions and in the absence of phage, the QS deficient phenotype is selected. However, regardless of the initial proportion of QS-deficient mutant used (10 or 50%), the addition of bacteriophage JBD30 selected the wild-type QS phenotype and limited social cheating (**Figure 2** and Supplementary Material, Figures SM1 and SM2 in the Data Sheet/Mathematical Models). Corroborating our experimental results, the mathematical model showed that the net outcome with respect to the balance between the *lasR rhlR* mutant and wild-type depends not only on public goods production and the level of protection against phages but also on the phages concentration which dynamically changes over time (Supplementary Figure SM2). In addition, experimental tests performed with D3112 showed a similar trend (data not shown).

### QS-Proficient Strain Lysogenized with JBD30 Is Less Prone to Be Exploited by QS-Deficient Mutants

**FIGURE 3:**
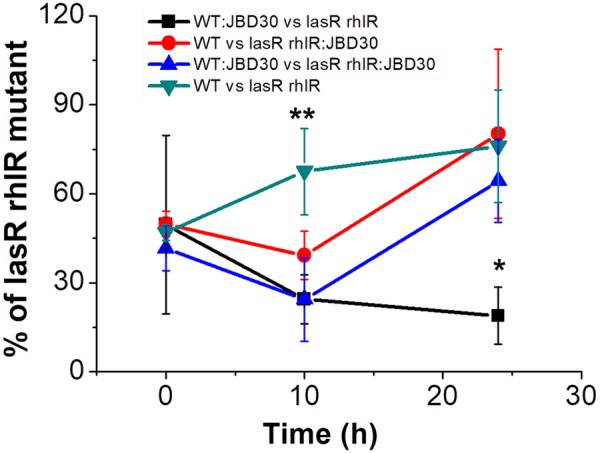
Lysogenic wild-type strain for the phage JBD30 restricts the survival of *lasR rhlR* mutants in M9 caseinate medium. Competitions were done in triplicate (averages are shown). WT:JBD30 and *lasR rhlR*:JBD30 correspond to the lysogenic versions of JBD30 for the strains indicated. The outcome of the competition of WT lysogenized by JBD30 is different from the outcomes of the other three competitions at 24 h (^∗^), *P* < 0.05 in a two-tailed Student’s *t*-test, and the proportion at 10 h of the competition without lysogens was different from all the other competitions at the same time (^∗∗^) *P* < 0.05 in a two-tailed Student’s *t*-test.

Due to the temperate nature of the phages used in the experiments, we decided to explore the impact of lysogeny on the QS selection by making competitions using lysogenic populations. When lysogenic clones of the wild-type (WT:JBD30) strain infected by JBD30 were competed against the non-lysogen *lasR rhlR* mutant, the presence of the phage carried by the wild-type host allowed it to significantly decrease the number of QS deficient mutants in caseinate medium at 24 h. In contrast, when the phage was carried by the mutant strain (*lasR rhlR*:JBD30) and when the phage was carried by both strains (WT:JBD30 vs *lasR rhlR*:JBD30), no differences were found relative to the competition in the absence of a lysogenic strain (WT vs *lasR rhlR*) after 24 h; i.e., when there was no phage present (**Figure 3**).

### Selection of Strains with an Active QS System by D3112 and JBD30 Bacteriophages *in Vivo*

**FIGURE 4:**
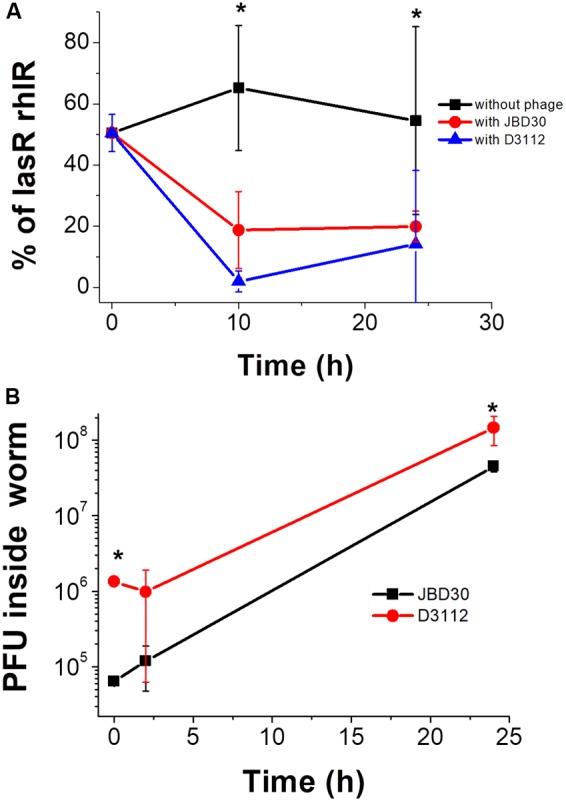
**(A)** *In vivo* selection of the QS phenotype by the addition of D3112 or JBD30 phage during a *Galleria mellonella* infection with the wild-type and *lasR rlhR* mutant presented as a 50% initial mixture. Experiments were done in four independent cultures, and the average is shown. **(B)** Phage replicates inside the infected worms. Experiments were done counting the pfu in three independent worms per phage (averages are shown). The differences between the proportion of the mutant without and with either phage at 10 and 24 h are significant except for D3112 at 24 h, *P* < 0.05, in a two-tailed *t*-test (^∗^), as well as the increase of either phage at 24 h, *P* < 0.05, in a two-tailed Student’s *t*-test.

After demonstrating the *in vitro* QS selection by the two phages, D3112 and JBD30, their effect *in vivo* was evaluated using the *G. mellonella* infection model. For these experiments, a mixture of 50% wild-type and *lasR rhlR* mutant (∼50 total cfu) was inoculated into the larvae, and immediately after infection, either phage D3112 or JBD30 was added to the larvae (or 0.9% NaCl sterile solution as negative control). Hemolymph samples were taken at 10 and 24 h after the addition of the phage and the number of bacteria and phage were determined. The percentages of both strains as a function of time revealed that the addition of these phages strongly selected for the presence of the QS system *in vivo* (**Figure 4A**). In addition, the plaque forming units (pfu) determination from the hemolymph (at the beginning, 2 and 24 h after phage addition) confirmed an active phage infection inside the larvae since phage titers increased (**Figure 4B**).

### QS Selection by Bacteriophage Increases Virulence with *G. mellonella*

Since QS enhances *P. aeruginosa* virulence in diverse animal models (Wu et al., 2004; Papaioannou et al., 2013; Castillo-Juárez et al., 2015) and we found that bacteriophages select for active QS systems *in vivo* (**Figure 4A**), we decided to test if this selection could also increase virulence toward *G. mellonella*. As expected, a 100-fold lower initial inoculation of the wild-type strain compared to the QS mutant was required to yield similar death curves (Supplementary Figure S2), corroborating that the wild-type strain was much more virulent than the mutant. Then, when we inoculated a 50% mixture of both strains (∼8 cfu, determined by cfu counting), and 8 h after infection, phage JBD30 (∼4 × 10^5^ pfu) or saline solution, were added, and we found that larvae death was accelerated by the addition of the bacteriophage (Supplementary Figure S3). Hence, the *in vivo* selection of QS by JBD30 can be related to the increased bacterial virulence.

### JBD30 Phage Preferentially Attach to the *lasR rhlR* Mutant

Since previous studies had shown that in *E. coli* and *V. anguillarum* QS proficient strains downregulate phage attachment at high cell densities (Hoyland-Kroghsbo et al., 2013; Tan et al., 2015; Hoque et al., 2016), we performed phage attachment assays for both the wild-type and QS-mutant strains using JBD30. Our results showed that the phage attached preferentially to the QS mutant in LB only at high density (OD 600 nm ∼2.2) with 76 ± 3.3 and 57 ± 3.6% of the phage adsorbed by the mutant and wild-type, respectively (Supplementary Figure S4A). In contrast, preferential attachment to the QS mutant was not observed in LB low density cultures (OD 600 nm ∼0.5) reinforcing the idea that QS mediates the decrease in phage adsorption observed in the wild-type strain (Supplementary Figure S4B).

## Discussion

Our results demonstrate that bacteriophages are able to select strains with functional QS systems and show that phage interactions with QS-deficient cells produce negative effects for both phage replication and bacterial survival. Recent reports in different bacterial species show that QS can control anti-phage defense mechanisms leading to lower susceptibility to phage infection in QS-proficient cells (Aaron et al., 2004; Tan et al., 2015; Hoque et al., 2016). In such scenarios, the selection of functional QS systems could be possible due the reduced ability of the phage to infect cells carrying active systems (although this has not been experimentally explored yet); however, we observe the opposite behavior in our model; i.e., phages infect more efficiently the wild-type strain as compared to the QS-mutant. The preferential attachment of the JBD30 phage to the QS mutant may be due to a higher expression of the phage receptor as it has been observed for *V. anguillarum* (Tan et al., 2015). The receptor in the case of D3112 phages (including JBD30) is the type IV pilli (Tfp) (Wang et al., 2004). However, it has been reported that Tfp expression in the *lasR rhlR* mutant of the PAO1 strain is similar to the expression in the wild-type (Beatson et al., 2002), and we found that Tfp-dependent twitching motility was the same for the PA14 wild-type strain and the QS-mutant (data not shown). Hence, the higher susceptibility of the QS-deficient strain for phage adsorption is likely not due to a higher Tfp expression. Other specific molecular phenomena, such as the higher synthesis of QS-controlled capsule components, might be a factor in decreasing phage attachment toward QS proficient individuals. In agreement with this hypothesis, the surface hydrophobicity of the wild-type strain is much higher than that of the mutant and it decreases by the treatment of alginate lyase (data not shown). Whether this feature is related to the phage adsorption rate or QS selection observed in our experiments has yet to be tested.

Due to the temperate nature of the phages used in our experiments, lysogeny represents an alternative explanation for the selection of QS systems. It was recently reported that temperate LES phages can increase the competitiveness of *P. aeruginosa* lysogens against their non-lysogenic version (Davies et al., 2016). In that study, the authors demonstrate that lysogens invade the phage-susceptible population during competition experiments in a rat chronic lung infection model (Davies et al., 2016). Our findings add to this result by showing that during infection, temperate bacteriophages may maintain cooperative behavior by eliminating QS-deficient social cheaters that lack the phages. In contrast, if the QS-deficient mutant contains the temperate phage at the beginning of the competition with a non-lysogenic wild-type host, the proportions at 24 h do not significantly change relative to the conditions without phage. As expected, when both strains carried the phage from the beginning of the competition, the mutant proportions increased at 24 h since the QS mutant is now protected from phage attack and can exploit the wild-type as in the no phage control (**Figure 3**). Since our results indicate that phage infection is more efficient in the QS-proficient strain, it is likely that a greater number of lysogens is produced in this genetic background compared to the mutant version. Hence we speculate that a greater rate of wild-type lysogenization and phage production compromise the survival of the QS-defective strain during *in vitro* and *in vivo* competitions, thus leading to the selection of QS active systems. The mechanisms affecting the phage production in the QS-deficient strain remain to be elucidated; yet, it is intriguing to observe such behavior in spite the phage preference for adsorption to the mutant cells. These results might imply a phage dependence on QS-regulated molecular mechanisms for its optimal replication or reflect the fragility of the QS defective bacteria in sustaining phage infections.

In addition to maintaining active QS systems, our results with bacteriophages also have important implications for the effectiveness of compounds used to reduce pathogenesis by masking QS; i.e., quorum-quenching (QQ) compounds. First, our results agree with the observation that in the *P. aeruginosa*, QS-defective population, i.e., in the *lasR rhlR* mutant that mimics a strain that has QS inhibited completely when QQ compounds are used, these cells are much more sensitive to environmental stress than the wild-type strain with its functional QS system (García-Contreras et al., 2015; García-Contreras, 2016). This has been shown for heat shock, heavy metal exposure and oxidative stress, and the addition of H_2_O_2_, all of which select the QS-proficient phenotype during growth on casein as the sole carbon source (García-Contreras et al., 2015). Given that bacteriophages are omnipresent in the natural environments of bacteria (Chibani-Chennoufi et al., 2004), we propose that broad sources of environmental stress in addition to spatial structure could promote the selection of QS-proficient bacteria (García-Contreras et al., 2016); i.e., the QQ resistant population, when QQ compounds are used. Critically, our results with bacteriophages demonstrate that although cheating may prevent the selection and spread of QS-proficient individuals (i.e., those bacteria resistant to QQ compounds) under ideal growth conditions (Mellbye and Schuster, 2011; Gerdt and Blackwell, 2014), the higher susceptibility of QS-deficient mutants in the presence of bacteriophages allows the selection and maintenance of a functional QS system; i.e., the presence of bacteriophages, promotes selection of QQ resistant bacteria.

The current global antibiotic resistance crisis has generated a resurgence of interest in phage therapy in Western medicine (Young and Gill, 2015). Young and Gill (2015) in pointed out that a deeper ecological understanding of bacteria-phage interactions is a requirement to develop and establish successful phage therapy treatments (Young and Gill, 2015). Since many virulence factors are QS controlled, exploring the role of QS is the obvious next step in advancing this goal. To date, it is known that bacterial isolates from chronic infections tend to show genetic variability within the same host (Wilder et al., 2009). In *P. aeruginosa* infections, *lasR* mutants accumulate accompanied by a lowered mean virulence (Ciofu et al., 2010; Hogardt and Heesemann, 2013). Critically, a certain, albeit sometimes small fraction of strains with intact genes seem to always remain (Wilder et al., 2009). CF patients with chronic *P. aeruginosa* infections are prone to periods of severe exacerbation, for which the cause yet remains unknown. New infections by more virulent *P. aeruginosa* strains and the influence of temperate phages have been dismissed as possible causes (Aaron et al., 2004; James et al., 2015). Reports about negative effects during clinical phage therapies are rare, but exist (Sulakvelidze et al., 2001). In view of our results, the shifts in virulence may be due to the complex interactions of the pathogen with its phages. Our results also suggest that a strategy to cope with the undesired rise of highly virulent strains after phage addition should be considered when using QS inhibition, for example by testing if the phages alone or in combination (either lytic or temperate) select QS proficient or deficient strains or otherwise do not have any bias in order to avoid the potential risks of selecting more virulent QS proficient strains.

Although lytic phages are the recommended type of viruses for therapeutic purposes, the use of temperate phages has been also proposed under certain circumstances (Chung et al., 2012). Our results suggest that even in such circumstances the phage-driven selection of QS active systems could represent a previously unexplored risk.

**FIGURE 5:**
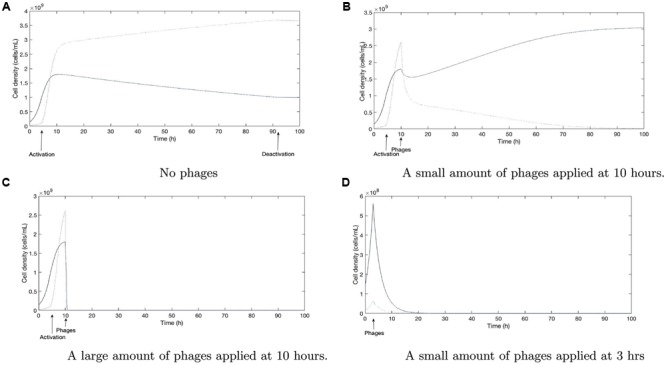
Numerical simulations of the model (2): the solid line corresponds to the wild-type population and the dotted one to the QS-deficient double mutant. Parameter values used to produce this simulation (values of β*i* for *i* = 1,…,9) can be found in Remark 1 of the Supplementary Material. **(A)** Without phage therapy, a cheater that does not produce QS controlled exoproducts has a growth advantage over the wild-type population leading to mutant invasion. **(B)** With the same parameter set, adding a certain amount of phages after induction, results in an increase of the relative fitness of the wild-type—compared to the cheater. This increase of the percentage of the higher virulent wild-type is a potential risk to the host. **(C)** Applying a higher dose of phages after QS induction can eventually completely eradicate the bacteria. **(D)** Eradication is also possible when phages are applied before QS is induced.

We further evaluated the potential consequences of our results in regard to phage therapy under such conditions of genetic variability by extending our mathematical model (see Mathematical Modeling Supplement) to include long-term effects in the host. We performed a series of numerical simulations, first assuming host colonization with an initially low fraction of cheaters (10%). Without phages and with a carbon source that requires shared resources, cheaters will begin to out-compete the wild-type due to exploitation of public goods once the latter has reached the QS activation threshold (**Figure 5A**). Phage therapy will usually start at some time after the patient has developed symptoms of infection; i.e., after induction of QS-induced virulence factors. This can result in the low-virulence, QS mutants being out-competed and an increase of absolute number of wild-type cells, depending on the strength of the interactions (**Figure 5B**). The fitness benefit of the cheater due to utilization of public goods will probably be lower in the host than in batch culture experiments, as the spatial structuring of bacterial cells (e.g., in the lung) will dampen it (Lam et al., 1980; Nadell et al., 2010). The model is able to display successful therapy, but only after a large dose of phages is applied after activation (**Figure 5C**). A possible strategy to avoid the undesired promotion of highly virulent strains would be to start phage therapy before the onset of QS induction (**Figure 5D**). However, this may be difficult to implement in practice as the infection yet remains undetected due to the absence of severe symptoms. Note that parameters determining the net outcome (see model), such as costs of public good production (β_2_) and initial phage concentration [V(T_P_)], can change over time or in dependence on the specific environmental conditions (Mellbye and Schuster, 2014). Additionally, a variety of anti-phage strategies may exist in bacteria. In summary, a patient-specific prediction of the net outcome and thus a quantification of the risk, would be highly complex. We remark here that spatial structuring of the bacterial population probably also impedes complete eradication even if high doses of phages are applied, as phage pressure on cells in deeper layers of colonies and biofilms will be limited (Abedon, 2016). Overall, we predict that phage therapy may constitute a hidden risk of exacerbating the infection by selecting for more virulent strains. Alternatively, a combined application of QS inhibitors and phages can be considered.

Hence, more effort should be dedicated to test the effects of different types of phages in QS selection as well as characterizing the mechanisms involved in the selection of QS-proficient strains. For instance, we need to further investigate the higher susceptibility of the QS-deficient mutant to phage during competitions in spite of the fact that phage infection is more efficient in the wild-type strain. It is remarkable that phage production is reduced in the QS-defective background as compared to the QS-proficient cells; hence, lysis by the phages in the mutant may not always involve a successful infection process. In addition, for temperate phages, the lysogenic potential toward both strains should be taken into account since differences in this regard influence the competition outcome, as we have shown. The results presented here highlight the importance of determining the relative fitness of putative social cheaters not only under a single condition but by taking into account different environmental variables that can severely change the fitness of such cheats (O’Brien and Brockhurst, 2015; Wolf et al., 2015). We demonstrate that the presence of lysogenic bacteriophages act as a powerful driving force for the selection of functional bacterial QS systems both *in vitro* and *in vivo*, by stabilizing bacterial cooperation and therefore virulence.

## Materials and Methods

### Bacterial Strains, Phages, and Growth Conditions

The *P. aeruginosa* PA14 wild-type strain and the PA14 *lasR rhlR* mutant were provided by Dr. You-Hee Cho from the College of Pharmacy CHA University, South Korea (Park et al., 2005). Pre-cultures of both strains were grown in LB medium aerobically in flasks, at 37°C with 200 rpm shaking for ∼16 h. These pre-cultures were used to inoculate flasks with M9 minimal medium supplemented with 0.25% of sodium caseinate as the sole carbon source, and the cultures were grown under the same conditions. Growth was monitored by recording the turbidity (600 nm) with a spectrophotometer (UV-1800, Shimadzu). Bacteriophage D3112 was acquired from the Félix d’Hérelle Reference Center for Bacterial Viruses, Canada, and phage JBD30 was kindly provided by Dr. Alan Davison from the University of Toronto, Canada. Phage infection was tested on bacterial lawns of both strains by using the standard soft agar overlay method (Green and Sambrook, 2001) and recording the number of lytic plaques observed. The infection assays on liquid cultures were carried out on LB medium. PA14 and its derivative mutant were grown on 5 ml at 37°C with 200 rpm shaking for 10 h. At this point, 2 × 10^6^ pfu of phage JBD30 were added and the cultures were grown under the same conditions until 18 h where samples were taken to count the viable cells and phage produced.

### Competition Experiments

Cultures in M9 caseinate or M9 casamino acids medium were inoculated at an initial turbidity at 600 nm of ∼0.05 with different proportions of the PA14 wild-type and the QS mutant by mixing the pre-cultures and by growing under the conditions detailed above. Samples of each culture were taken at different cultivation times and were used to isolate colonies. The colonies were then transferred to LB plates with 3% skim milk to determine their exoprotease production to quantify the proportion of the two populations.

For analyzing the effect of bacteriophages on competitions between the wild-type and QS-deficient strains, we tested the temperate phages D3112 and JBD30. Approximately 2 × 10^3^ pfu of each phage were added at 4 or 8 h during the competition experiments, and subsequent samples were taken to estimate the proportion of the two populations.

Lysogens were obtained by exposing wild-type and *lasR rhlR* mutant liquid cultures to infection by phages JBD30 and D3112. Survivor colonies were obtained, purified, and tested for their phage production by spotting their cell-free supernatants on wild-type lawns, and for their immunity against phages JBD30 or D3112. In addition, PCR reactions were performed by using primers (F: 5′-GATACCTGACCCGCAACGG-3′ and R: 5′-AGATGCCGATGGGGATCAGT-3′) targeting a conserved region of the D3112 and JBD30 genomes to confirm the presence of the phages in the bacterial genome. Competitions using these lysogens were done as explained above for non-lysogenic bacteria.

For the *in vivo* competition experiments, cells were taken from LB overnight cultures and mixtures of approximate 1:1 ratio of the wild-type and *lasR rhlR* mutant were made, then the mixtures were diluted with 0.9% sterile NaCl solution and ∼50 viable bacteria of each mixture (estimated by cfu determination) were injected into the larvae, in the absence and presence of either D3112 or JBD30 phage, which were administrated immediately after the bacteria by injecting them diluted in 0.9% sterile NaCl at ∼9 × 10^5^ and ∼6 × 10^4^ pfu per larvae, respectively. Samples of the hemolymph (∼3 ml) were taken after 10 and 24 h to determine the proportion of wild-type and mutant strain, and after 2 and 24 h of inoculation to determine the number of phages.

### Virulence Test

The virulence of individual strains and strain mixtures against *G. mellonella* (last instar larvae) was tested by injecting different dilutions (made in 0.9% sterile NaCl solution) of LB cultures of wild type and *lasR rhlR* mutant strains using overnight LB cultures, and the approximate number of bacteria inoculated was estimated by cfu determination and virulence was evaluated by assessing *G. mellonella* survival daily. Experiments were done using at least 10 larvae per condition.

### Phage Adsorption

Phage adsorption of JBD30 to the wild-type and mutant strains was tested on cultures at the logarithmic and stationary phase by following a modified version of a previously reported protocol (Roncero et al., 1990). Cells in the logarithmic and stationary phase were taken at turbidity at 600 nm of ∼0.5 and ∼2, respectively. Cells taken from both growth phases were adjusted to turbidity of 2.2, and 200 μl were mixed with an equal volume of modified phage buffer (50 mM Tris–HCl pH 8, 10 mM MgSO_4_, 100 mM NaCl, and 0.01% gelatine) containing 1 × 10^4^ pfu. CaCl_2_ (5 mM) was added to the mix, and it was incubated for 10 min at 37°C. Bacteria were removed from the mix by centrifugation at 9300 *g* for 15 min, and the supernatants were treated with chloroform. Phage was quantified using the soft agar overlay method with bacterial lawns of the PA14 strain. Reduction in the initial number of pfu was considered as the fraction of viral particles bound to the bacterial cells.

### Surface Hydrophobicity

This phenotype was determined by estimating the percentage of cells transferred from an aqueous to a hydrophobic hexadecane (Sigma) phase using overnight LB cultures (García-Lara et al., 2015) with and without alginate lyase (Sigma) at 1 mg/ml.

### Statistical Analysis

All experiments were done at least in triplicate; values are expressed as mean ± SD. Statistical significance for **Figures 1–4** were evaluated by a two-tailed Student’s *t*-test. Plots and their analysis were done using the SPSS and the Origin 8.0 software.

### Mathematical Modeling

Two mathematical models were developed, each consisting of sets of ordinary differential equations. The first one focused in estimating the impact of QS-regulated phage protection on the relative fitness of the wild-type strain relative to the QS-deficient strain. The purpose of the model was first to test the validity of the conclusions from the competition experiments by comparing the time course obtained in experiments with the model’s solutions obtained numerically (solving the equations); i.e., simulations, and second to get a rough estimation about the strength of these fitness effects. For more details, see mathematical modeling supplement. This model involved time-dependent parameters. The second model was developed to explore the long-term effect in the host. To this end, an equation to explicitly account for the dynamics of the phages was added. This model involved density-dependent parameters to account for QS-based activation. The details of the modeling framework of both models can be found in the Supplementary Material.

## Author Contributions

MS-M, PC-T, AC, DC, WF, MC, BP-E, NC, and YN performed the experiments. JP-V and BH made the mathematical modeling. AC, JP-V, BH, GG, TM, TW, and RG-C conceived the study. AC, JP-V, BH, TM, TW, and RG-C wrote the manuscript. JB-D and PC-T analyzed the data.

## Conflict of Interest Statement

The authors declare that the research was conducted in the absence of any commercial or financial relationships that could be construed as a potential conflict of interest.
