# Supplementary material for: Selection of Functional Quorum Sensing Systems by Lysogenic Bacteriophages in Pseudomonas aeruginosa
Source: Front Microbiol. 2017 Aug 31;8:1669. doi: 10.3389/fmicb.2017.01669 (PMC5583629; doi:10.3389/fmicb.2017.01669)
Supplement: Supplementary file 1 [file Presentation_1.PPTX]

## Slide 1
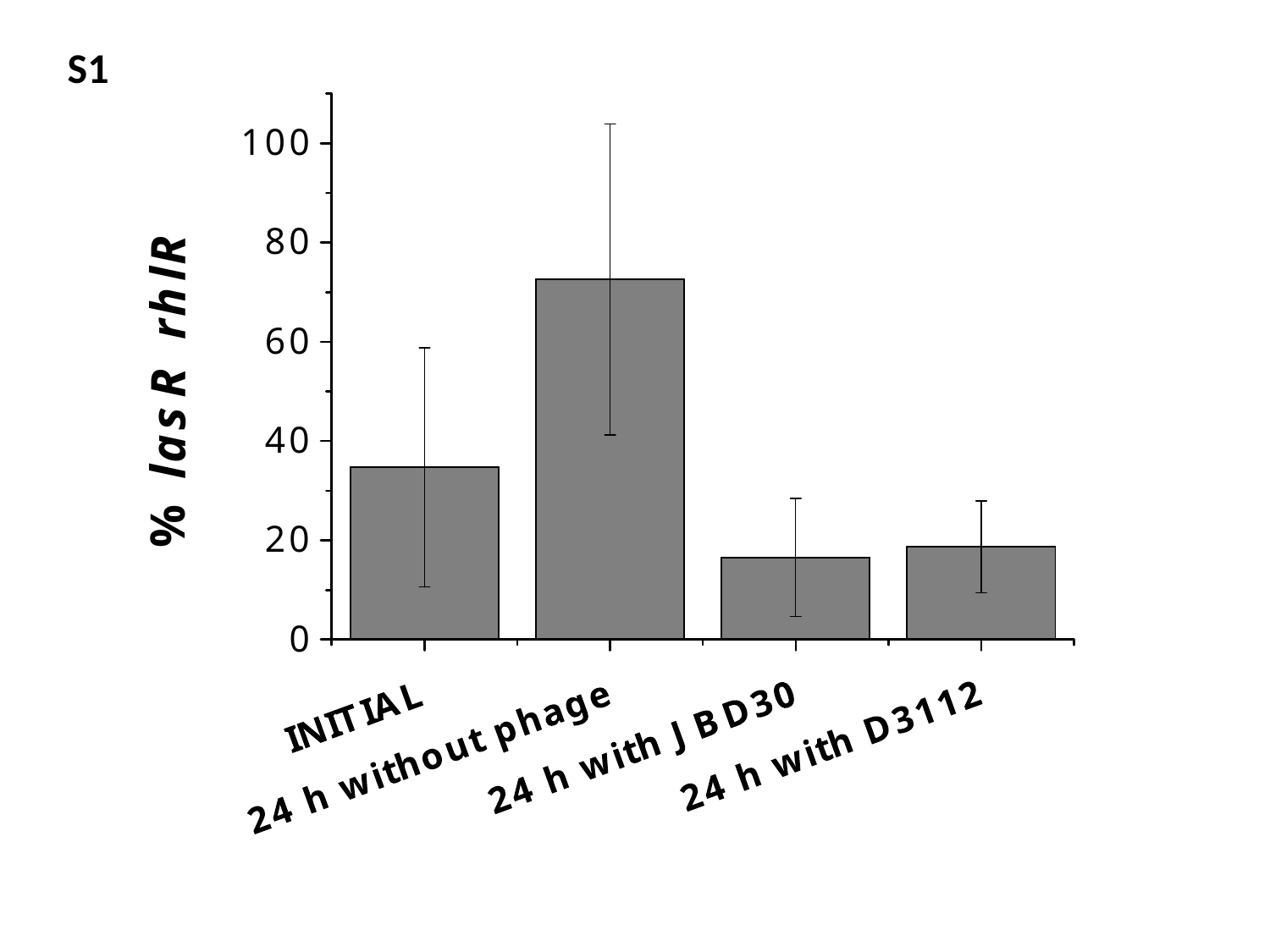

S1

## Slide 2
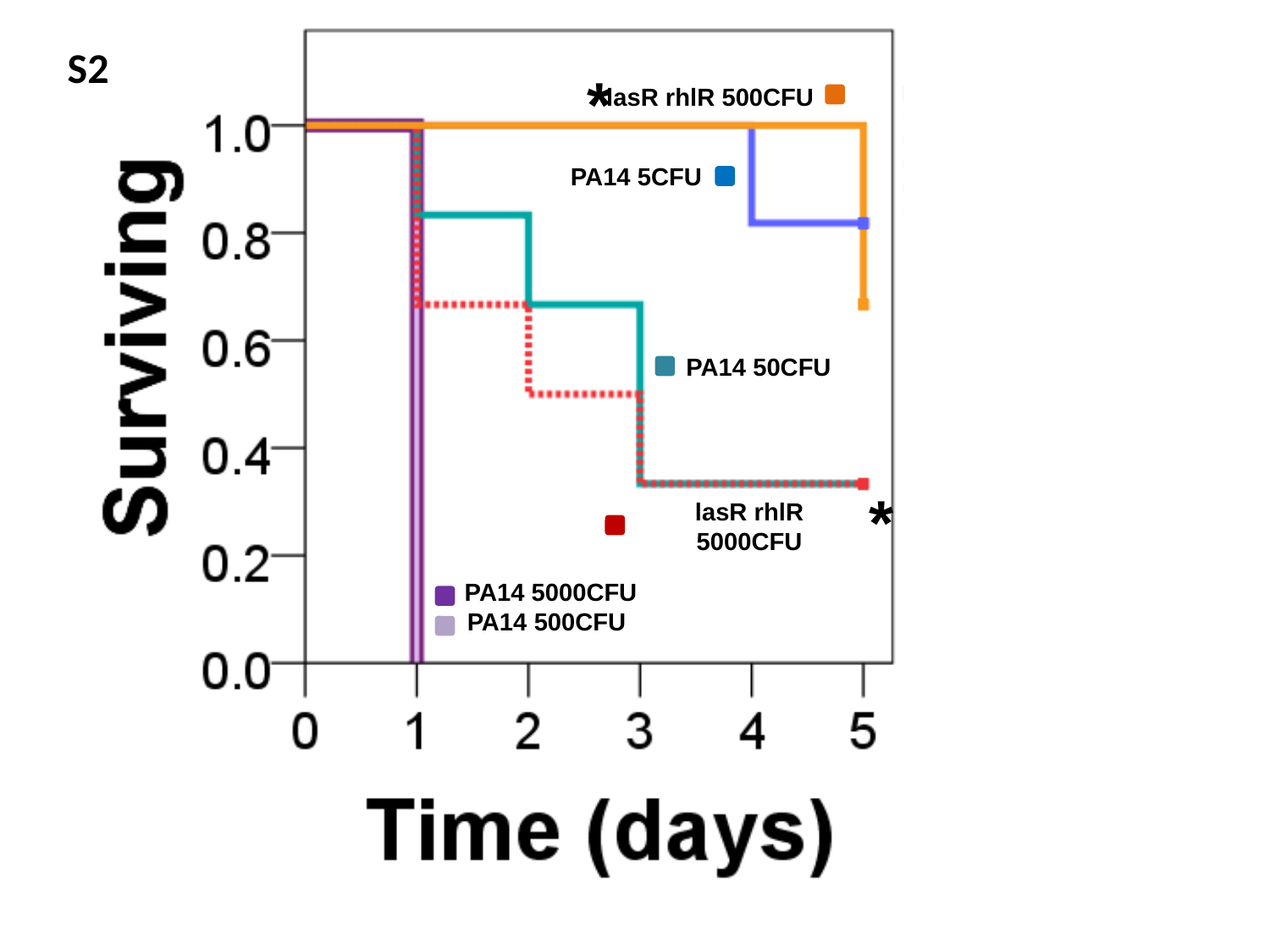

lasR rhlR 500CFU
*
PA14 5CFU
PA14 50CFU
*
lasR rhlR 5000CFU
PA14 5000CFU
PA14 500CFU
S2

## Slide 3
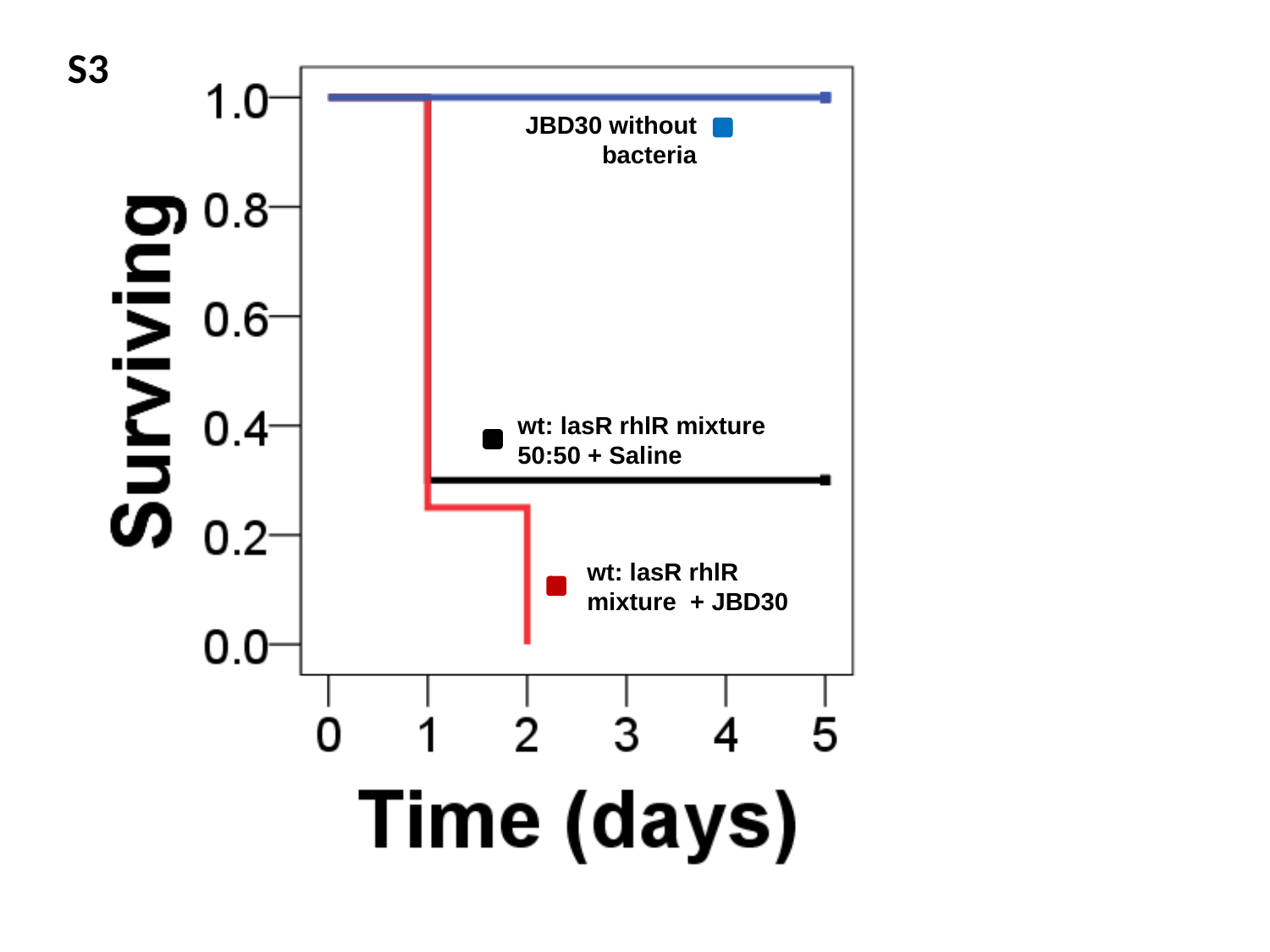

S3
JBD30 without bacteria
wt: lasR rhlR mixture 50:50 + Saline
wt: lasR rhlR mixture + JBD30

## Slide 4
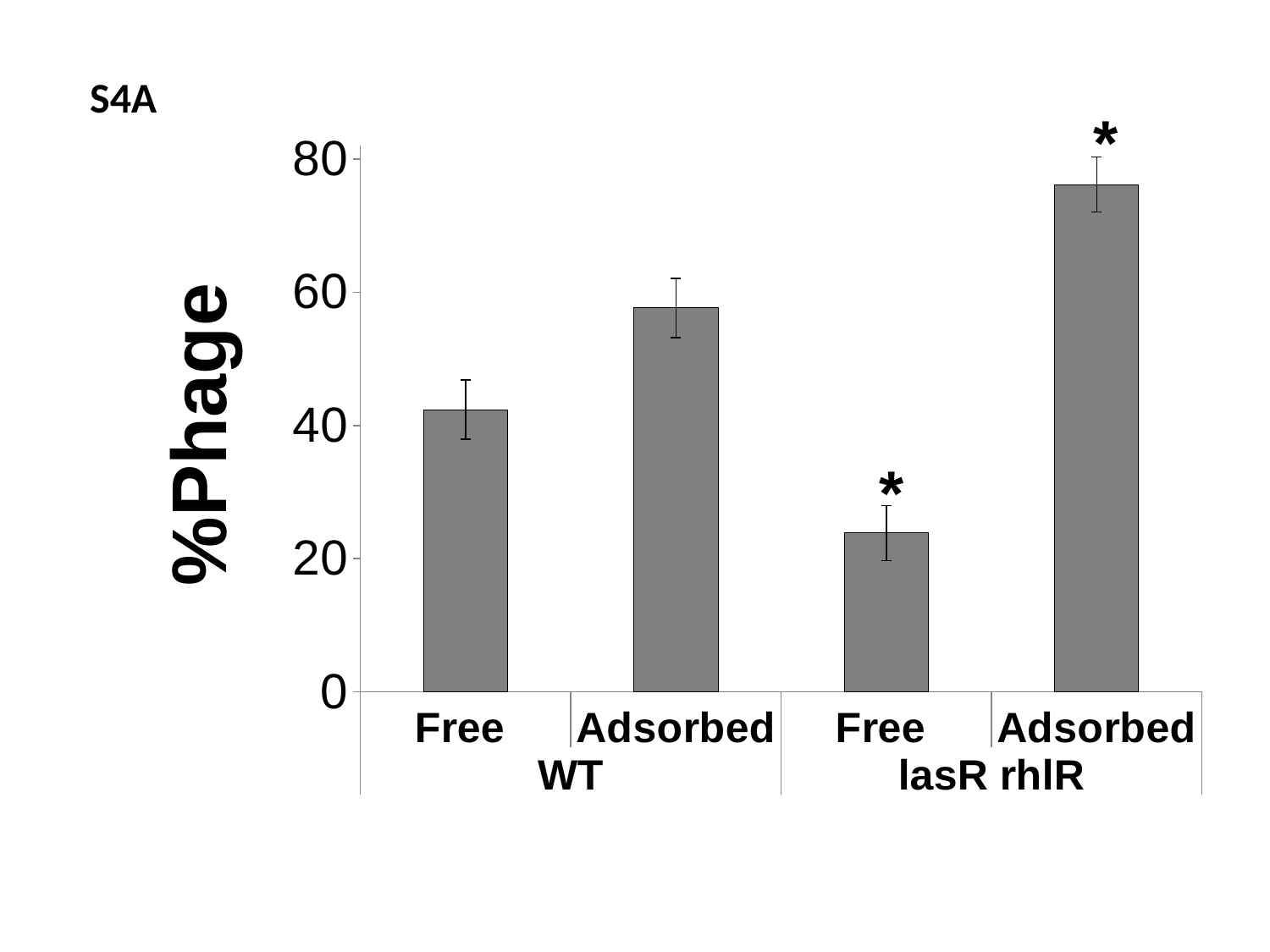

S4A
### Chart
| Category | |
|---|---|
| Free | 42.368863025359325 |
| Adsorbed | 57.631136974640675 |
| Free | 23.83926001551987 |
| Adsorbed | 76.16073998448013 |*
*

## Slide 5
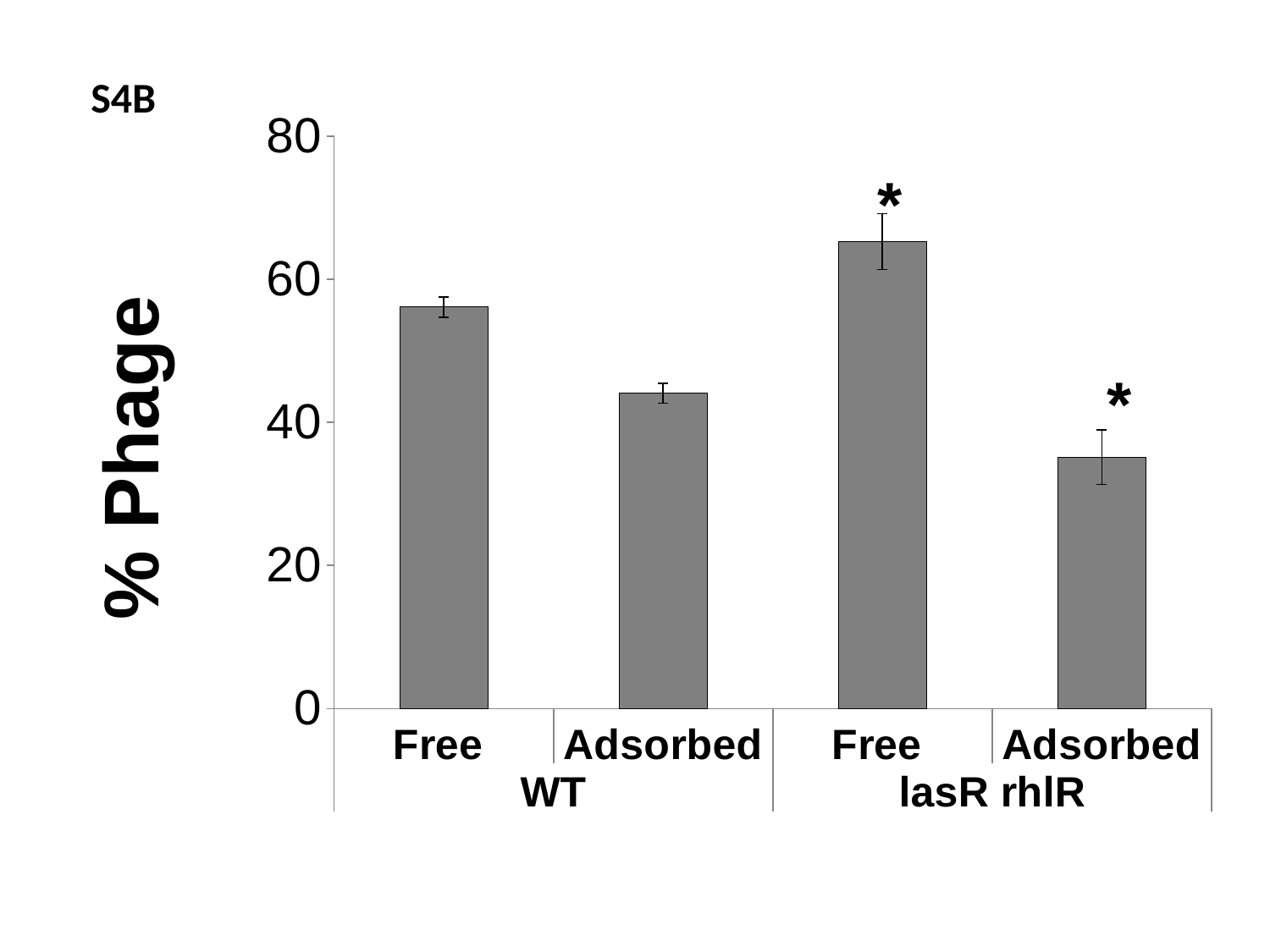

S4B
### Chart
| Category | |
|---|---|
| Free | 56.092987488443924 |
| Adsorbed | 44.0320741769413 |
| Free | 65.22953238932871 |
| Adsorbed | 35.135435253653434 |*
*

## Slide 6
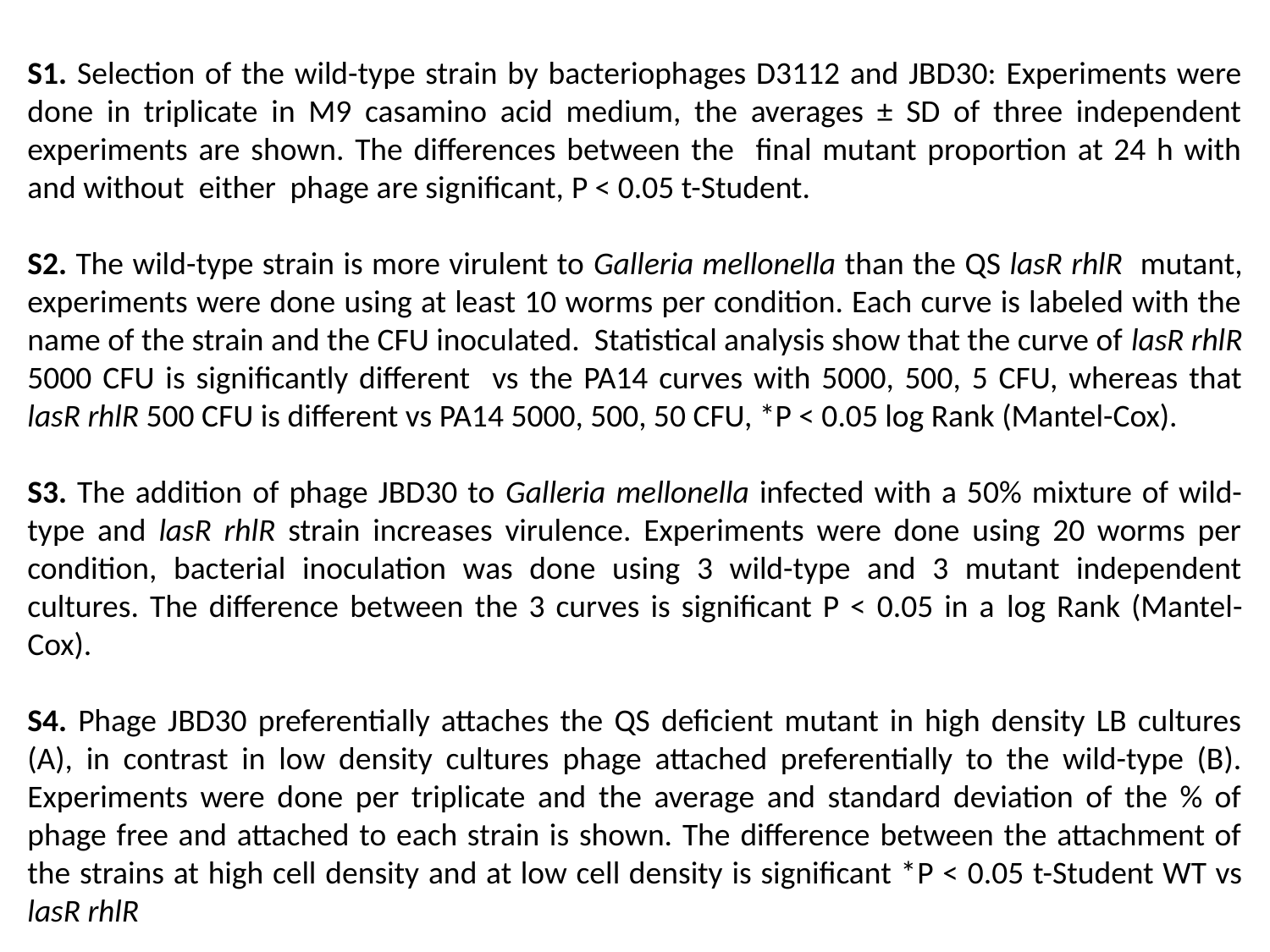

S1. Selection of the wild-type strain by bacteriophages D3112 and JBD30: Experiments were done in triplicate in M9 casamino acid medium, the averages ± SD of three independent experiments are shown. The differences between the final mutant proportion at 24 h with and without either phage are significant, P < 0.05 t-Student.
S2. The wild-type strain is more virulent to Galleria mellonella than the QS lasR rhlR mutant, experiments were done using at least 10 worms per condition. Each curve is labeled with the name of the strain and the CFU inoculated. Statistical analysis show that the curve of lasR rhlR 5000 CFU is significantly different vs the PA14 curves with 5000, 500, 5 CFU, whereas that lasR rhlR 500 CFU is different vs PA14 5000, 500, 50 CFU, *P < 0.05 log Rank (Mantel-Cox).
S3. The addition of phage JBD30 to Galleria mellonella infected with a 50% mixture of wild- type and lasR rhlR strain increases virulence. Experiments were done using 20 worms per condition, bacterial inoculation was done using 3 wild-type and 3 mutant independent cultures. The difference between the 3 curves is significant P < 0.05 in a log Rank (Mantel-Cox).
S4. Phage JBD30 preferentially attaches the QS deficient mutant in high density LB cultures (A), in contrast in low density cultures phage attached preferentially to the wild-type (B). Experiments were done per triplicate and the average and standard deviation of the % of phage free and attached to each strain is shown. The difference between the attachment of the strains at high cell density and at low cell density is significant *P < 0.05 t-Student WT vs lasR rhlR
