# Supplementary material for: Selection of Functional Quorum Sensing Systems by Lysogenic Bacteriophages in Pseudomonas aeruginosa
Source: Front Microbiol. 2017 Aug 31;8:1669. doi: 10.3389/fmicb.2017.01669 (PMC5583629; doi:10.3389/fmicb.2017.01669)
Supplement: Supplementary file 2 [file Data_Sheet_1.PDF]

Mathematical model of QS-mediated phages protection

The experiments presented in the main text indicated that QS induces protection in *Pseudomonas aeruginosa* against phage-induced lysis. Following the experiments, we developed a simple mathematical model to describe how the phage impacts on the evolutionary fitness of mixed bacteria populations of *P. aeruginosa* wildtype ($PA14$) and its $lasR\ rhlR$ double mutant. First, our aim was to test whether the mechanistic conclusions drawn from the experiments about this influence are suitable to qualitatively reproduce the experimental dynamics, i.e. to validate these conclusions using a mathematical model. Secondly, we aim to obtain a rough estimation for the strength of these fitness effects. Note that a specific representation of each interaction step at the molecular level was not the object of this model, as this indeed it is not possible based on the actual state of knowledge.

Model I framework

We will use the following variables for each population type:

- $PA14$ wt strain (we will denote by $R(t)$, units cells/mL)
- Double mutant $lasR\ rhlR$ (we will denote by $S(t)$, cells/mL)

and the associated parameters, found in table SM0, where we denote in grey cells QS-regulated parameters. The ODEs system is formulated as:

$$\dot{R} = (\beta_1 - \beta_2)\left(1 - \frac{R(t)+S(t)}{\beta_3}\right)\left(1 + \frac{R(t)\beta_4}{R(t)+S(t)}\right)R(t) - (\beta_5 - \beta_6)R(t) \tag{1a}$$

$$\dot{S} = \beta_1\left(1 - \frac{R(t)+S(t)}{\beta_3}\right)\left(1 + \frac{R(t)\beta_4}{R(t)+S(t)}\right)S(t) - \beta_5 S(t) \tag{1b}$$

To build the mathematical model, we focused on the following assumptions:

- Logistic growth of bacteria; with basic growth rate β_1 describing the growth without QS induction. Both population types, namely S and R, have the same basic growth rates.
- QS induction causes costs (β_2), e.g. for production of public goods and/or private goods, only the wildtype pays this cost.
- There is a carrying capacity, i. e. a cell density saturation level imposed by the nutrients limitation in the media (β_3)
- QS induced production of public goods provides benefit to both wildtype and cheater cells (represented by the additional growth rate β_4)
- Phages cause bacterial death, with a death rate (β_5).
- QS induces some protection against phage-related death rate (β_6). Only QS proficient bacteria are protected (private good).
- Bacterial population growth is the slowest process covered by the model, i.e. the other processes like QS induction of private and public good are in equilibrium
- Homogeneously mixed populations, i.e. no diffusion effects.
- Processes like emerging resistance in the bacteria or lysogeny are too slow to be of significant relevance during the experiments.

β_1 growth rate	β_2 growth cost for doing QS
β_3 carrying capacity	β_4 QS benefit by public goods
β_5 death rate by phages	β_6 QS-regulated phages protection

Table SM0: Parameters. The units of $\beta_1, \beta_2, \beta_5$ and β_6 are h^{-1}, units of β_3, S and R are $cells/mL$ and β_4 is dimensionless. Parameters in grey cells are QS-regulated.

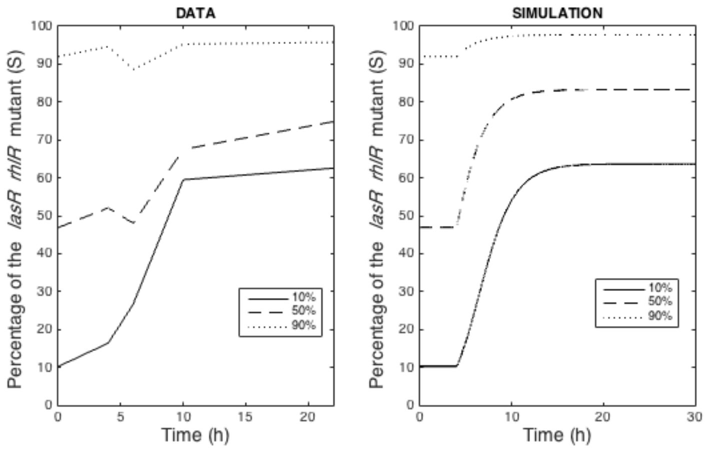

Figure SM1: Simulations (right) of model (1) versus the data from experiments (left). Here all competition cases (initial percentage of cheaters) are shown. Parameters used: $\beta_1 = 0.5, \beta_2 = 0.4, \beta_3 = 4.7 \times 10^9, \beta_4 = 1.0, \beta_5 = 0.28, \beta_6 = 0.23$.

1 Numerical solutions and results

We solve system (1) numerically using the appropriate initial conditions for each competition case. We chose parameters reflecting the qualitative behaviour of the competition experiments, with and without phages (see figs. SM1 and SM2, respectively).

Measurement of AHL concentrations over time (data not shown) in the experiments indicated that QS induction occurred roughly around $t = 4.0$ hours (data not shown). In accordance to this, we set up the parameter values as follows:

$$\beta_2 = \begin{cases} 0 & 0 < t < 4 \\ 0.4 & t \geq 4 \end{cases}, \quad \beta_4 = \begin{cases} 0 & 0 < t < 6 \\ 1 & t \geq 6 \end{cases}, \quad \beta_6 = \begin{cases} 0 & 0 < t < 6 \\ 0.2350 & t \geq 6 \end{cases}.$$

Note that while the effect of β_2 is assumed to coincide with activation time, this is not the case for β_4 or β_6, since we obtained generally best agreements between experimentally derived curves and simulations (fig. SM1 and SM2) if we assumed that the benefits of QS, i. e. public goods (β_4) and protection against phages (β_6) only starts at about $t = 6.0$ hours. A tentative explanation would be that a significant amount of benefit (e.g. nutrients) is available for the mutant cells only with a certain delay. This explanation fits to the experimental observation that in the 10% cheater cases (both with and without phages) the relative amount of cheaters increase after 4 hours, whereas there was even a small decline between $t = 4.0$ hours and $t = 6.0$ hours in the 50% case, and even a larger one in the 90% cases. This is inline with the hypothesis that after QS induction, first the producer cells benefit from their public good, and only after a short delay all cells benefit. If the fraction of wildtype is high, enough public good may be released so that the small fraction of mutants benefit earlier. Such an effect matches with the observation of privileged share in yeast [?, ?]. Note, however, our experiments were not designed for the privilege share aspect and other causes may explain the observed tendency (benefit delay with respect to activation time).

Our presented simulation thus ended up with 3 phases: a pre-QS and pre-phage phase (with $\beta_2, \beta_4, \beta_5$ and β_6 set to 0), a QS-induced pre-public good phase (with β_4 set to 0) and a fully induced phase when phages were added (all parameters are non-zero and positive in this phase).

In general, our simulations reflected well the time courses found in the experiments (figs. SM1 and SM2). Our results from the mathematical model support the notion that QS induced protection against phage lysis supports evolutionary stability of QS, and QS regulated cooperation via public goods.

The model is necessarily a simplification of reality. It focuses on one QS regulated public good and one private activity (although there are in fact a number of them, including pyocyanin), it ignores potential effects of changing environments on QS during the growth in batch cultures, and it assumes that the mentioned QS effect occur with a constant strength once initiated (ignoring that e.g. public goods accumulate over time). Furthermore, the measured data showed some standard deviation. The estimated parameters are thus just a rough estimation.

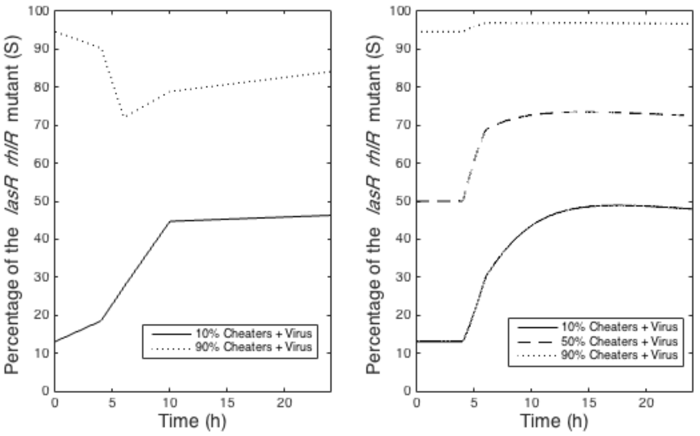

Figure SM2: Simulations (right) of model 1 versus the data (Phage D3112) from experiments (left) when phages were added. Here all competition cases (initial percentage of cheaters) are shown. Parameters used: $\beta_1 = 0.5, \beta_2 = 0.4, \beta_3 = 4.7 \times 10^9, \beta_4 = 1, \beta_5 = 0.28, \beta_6 = 0.23$.

2 Effect in hosts

We additionally aimed to estimate the longer term impact of QS induced phage protection during infections of *P. aeruginosa* in hosts. We therefore introduced an additional "death rate" for the bacteria β_7, reflecting e.g. effects of host defense, but also removal of bacteria from the host via e.g. feces. We furthermore consider the dynamics of the phages explicitly by extending model (1), resulting in:

Model II framework

Variables for each population type:

- $PA14$ wt strain (R)
- Double mutant $lasR\, rhlR$ (S)
- Phage (V)

and the set of equations:

$$\dot{R} = (\beta_1 - \beta_2(t)) \left(1 - \frac{R(t) + S(t)}{\beta_3}\right) \left(1 + \frac{R(t)\beta_4(t)}{R(t) + S(t)}\right) R(t) - \beta_5 R(t)V(t) + \beta_6(t)R(t)V(t) - \beta_7 R(t) \quad \text{(2a)}$$

$$\dot{S} = \beta_1 \left(1 - \frac{R(t) + S(t)}{\beta_3}\right) \left(1 + \frac{R(t)\beta_4(t)}{R(t) + S(t)}\right) S(t) - \beta_5 S(t)V(t) - \beta_7 S(t) \quad \text{(2b)}$$

$$\dot{V} = \beta_8 \left(\beta_5(R(t) + S(t)) - \beta_6(t)R(t)\right) V(t) - \beta_9 V(t). \quad \text{(2c)}$$

The associated parameters shown in Table SM2, including QS-regulated ones shown in grey.

Note that our model does not include lysogeny, in accordance with phage therapies which usually work with lytic phages. As the intermediate phase between induction of QS and supply of public goods described before (i.e. between $t = 4$ hrs and $t = 6$ hrs) is of minor relevance for the long term behavior, we omitted it here. As phages affect bacterial growth and thus QS induction, we use density-dependent QS activation and de-activation, i.e. ignore possible hysteresis [?, ?] effects for reasons of simplicity. In accordance with our experimental observations, we set the threshold $R_A = 1 \times 10^9$ cells/mL.

This is realized in following:

$$\beta_1 = 0.5, \beta_3 = 4.7 \times 10^9, \beta_7 = 0.01,$$

the activation-dependent parameters

$$\beta_2 = \begin{cases} 0 & R(t) < R_A \\ 0.4 & R(t) > R_A \end{cases}, \beta_4 = \begin{cases} 0 & R(t) < R_A \\ 3 & R(t) > R_A \end{cases}, \beta_6 = \begin{cases} 0 & R(t) < R_A \\ (1 - \frac{1}{6})\beta_5 = \frac{5}{6}\beta_5 & R(t) > R_A \end{cases},$$

β_1 growth rate	β_2 growth cost for doing QS
β_3 carrying capacity	β_4 QS benefit by public goods
β_5 phages dependent death rate	β_6 QS-regulated phages protection
β_7 phage-independent death rate	β_8 replication rate of phages
β_9 phages death rate	R_A activation density
T_P phages addition time	

Table SM2: Parameters. The units of $\beta_1, \beta_2, \beta_7$ and β_9 are h^{-1}, units of β_3, β_8, S and R are $cells/mL$, units of β_5 and β_6 are $mL/(cells \times h)$ and β_4 is dimensionless. Parameters in grey cells are QS-regulated. Grey cells denoted QS-regulated parameters.

and the phages-related parameters, which are dependent of the time phages were added, T_P.

$$\beta_5 = \begin{cases} 0 & 0 < t < T_P \\ 1 \times 10^{-4} & t > T_P \end{cases}, \beta_8 = \begin{cases} 0 & 0 < t < T_P \\ 1 \times 10^{-14} & t > T_P \end{cases}, \beta_9 = \begin{cases} 0 & 0 < t < T_P \\ 1 \times 10^{-15} & t > T_P \end{cases}.$$

Note that the choice of parameters values was based on experimental observations, such as the fact that phage JBD30 kills around 6 times the mutant than the wildtype. However, due to the lack of "in host" data for parameter estimation and the inevitable simplifications in our approach, the model meant to give rather a qualitative, not a quantitative prediction of what could happen.

3 Possible outcome of the use of phage therapy in the framework of QS-regulated phage protection

To investigate the possible long term implications of our experimental results for phage therapy against bacterial infections in hosts like humans, we display the following scenarios of emerging infections in which both wildtype bacteria and a small fraction of QS mutants are present:

With no phages present, See fig. SM3a.

Phages are added after onset of QS ($T_P = 10$ hrs), see fig. SM3b.

Phages are added at a later time at a high dosis (also 10 hrs): see fig. SM3c.

Phages are added early (at a high doses) successful therapy. See fig. SM3d.

Without phage therapy, a cheater that does not produce some QS controlled exoproduct can invade wild-type populations, consisting of cooperative individuals. that do produce the exoproduct. Note that the de-activation of QS takes place towards the end, resulting in a stable coexistence of wildtype and cheater (fig. SM3a). With the same parameter set, adding a certain amount of phages well after induction results, in the long term, in both, an increase of the relative fitness of the wildtype -compared to the cheater- (fig. SM3b) and an increase of the absolute number of wildtype cells (Fig. 5 in main text). In this case the therapy was unsuccessful, i.e. the bacteria are not eradicated. Applying a higher dosis of phages after QS induction can eventually completely eradicate the phages, however, there is an intermediate increase of the percentage of the higher virulent wildtype, which means a potential risk if some bacterial cells escape from eradication in the complex habitat (host), see fig. SM3c. Application of the phages before QS is induced avoids this problem as no accumulation of wildtype cells relative to cheater cells occurs, fig. SM3d.

Note that the outcomes shown on these simulations (for example eradication or failed eradication) heavily relies on the parameters values. These, in turn, depend on the host, the phages and the specific scenario in the patient. However, in each case, a certain risk of exacerbation of the infection by phage therapy cannot be excluded.

Remark 3.1 *To produce these simulations, the following parameters were used* $\beta_1 = 0.5, \beta_2 = 0.4, \beta_3 = 4.7 \times 10^9, \beta_4 = 3, \beta_5 = 1 \times 10^{-4}, \beta_6 = (1 \times 10^{-4})/7, \beta_7 = 0.01, \beta_8 = 1.0 \times 10^{-14}, \beta_9 = 1.0 \times 10^{-15}$*, the basic parameters were taken from simulations of Model I.*

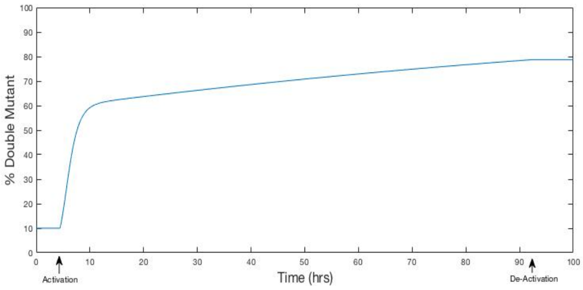

(a) No phages

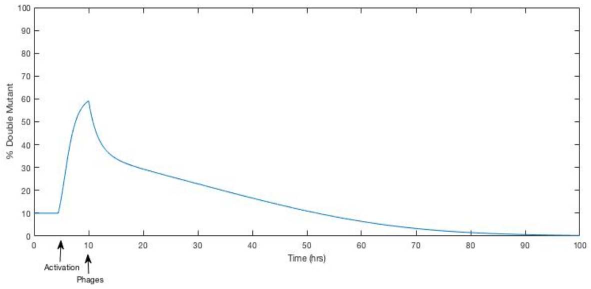

(b) A small amount of phages applied at 10 hrs

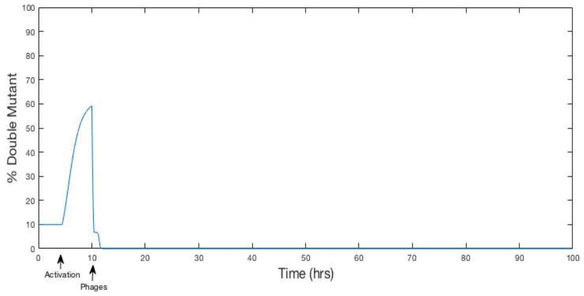

(c) A large amount of phages applied at 10 hrs

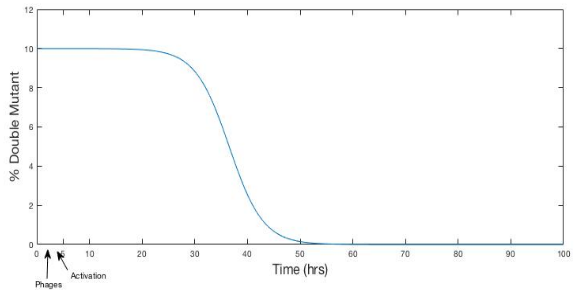

(d) A small amount of phages applied at 3 hrs

Figure SM3: Numerical simulations of model (2) displaying fractions of double mutant cells (a) After QS induction in wildtype cells, a cheater that does not produce some exoproduct can invade a wild-type population, consisting of cooperative individuals who do produce the exoproduct. Note that uninduction took place towards the end, resulting in a stable co-existence of wildtype and cheaters. Parameters values used to produce this simulation: amount of added phages: 0. (b) Phages added at $t = 10h$ after QS induction in wildtype cells, amount of added phages: 8000 $cells/mL$. (c) A large load of phages are added (after 4 hours) leading to bacterial population extermination. (d) A small load of phages are added early (before 4 hours) leading to bacterial population extermination. Parameters values used to produce these simulations (values of β_i for $i = 1, ..., 9$) can be found in Remark 3.1 of Supplementary Material, initial percentage of double mutant: 10%.
